# Fetomaternal outcome of pregnancy with Mitral stenosis

**DOI:** 10.12669/pjms.313.7020

**Published:** 2015

**Authors:** Nazia Ahmed, Hafeeza Kausar, Lubna Ali, Rakhshinda

**Affiliations:** 1 Dr. Nazia Ahmed, FCPS, Department of Obs/Gyn, Civil Hospital Dow University of Health Sciences, Karachi, Pakistan; 2 Dr. Hafeeza Kausar, MBBS, Department of Obs/Gyn, Civil Hospital Dow University of Health Sciences, Karachi, Pakistan; 3 Dr. Lubna Ali, FCPS, Department of Obs/Gyn, Civil Hospital Dow University of Health Sciences, Karachi, Pakistan; 4 Dr. Rakhshinda, FCPS, Department of Obs/Gyn, Civil Hospital Dow University of Health Sciences, Karachi, Pakistan

**Keywords:** Cardiac disease in pregnancy, Mitral stenosis, Valvular heart disease in pregnancy

## Abstract

**Objective:**

To evaluate the frequency of fetomaternal outcome of pregnancy with Mitral stenosis admitted in Civil Hospital Karachi.

**Methods:**

It was a two years descriptive study done in the Department of Obstetrics and Gynaecology Civil Hospital Karachi. All pregnant women with a known or newly diagnosed Mitral stenosis on echocardiography were included in the study. History was taken regarding age, parity, gestational age (calculated by ultrasound) and complaints. Mode of delivery and Maternal mortality noted. Foetal outcome was analyzed by birth weight and Apgar score.

**Results:**

A total of 101 patients meeting the inclusion criteria were enrolled in the study. The ages of the women ranged between 20-29 years (69%) and 81% were multigravidas. Vaginal delivery occurred in 67 (66.3%) women and 78.3% were term pregnancies. Preterm deliveries were 21.8% and 27.7% newborns were low birth weight. APGAR score <7 was found in 14.9% of neonates and 9 babies had intrauterine death. Low ejection fraction<55% was diagnosed in 20(13.9%) women and Maternal mortality was found in two cases.

**Conclusion:**

Heart disease in pregnancy is associated with significant morbidity, it should be carefully managed in a tertiary care hospital to obtain optimum maternal and foetal outcome.

## INTRODUCTION

The incidence of clinically significant cardiac disease during pregnancy has not changed for decades. The most recent studies report incidence of 0.1 to 4%.^1^ In developing countries like Pakistan, rheumatic heart disease still account for the majority cases and mitral stenosis is the most frequently observed valvular lesion.^2^ Extensive changes occur in the cardiovascular haemodynamics during pregnancy. Whilst these adaptations are well tolerated by the healthy women, those with heart disease can decompensate, resulting in significant morbidity and mortality.^3^ The cardiovascular changes occurring during pregnancy result in high flow, low resistance state changes which begin at 7 weeks of gestation and persist 2 weeks postpartum.^4^ Valvular heart disease is often recognized for the first time during pregnancy, when the pregnancy associated cardiovascular changes increase the demand on the heart and exacerbate symptoms like shortness of breath, palpitations, syncope, fatigability, and hemoptysis. Examination may reveal cyanosis, clubbing, raised JVP, cardiomegaly, murmurs, arrhythmias and basal crepts. The functional capacity of the heart is assessed by investigations such as electrocardiography, echocardiography, oxygen saturation and complete blood examination.^2^ Despite continuous improvements in diagnostic cardiology techniques, echocardiography remains the corner stone both for assessing the reversible physiological cardiac remodeling of pregnancy associated changes in valve patency or trans valvular flow pattern.^5^ In spite of prior lack of clinical symptoms, patients with acquired rheumatic heart disease, consisting mainly in stenosis of mitral and aortic valve, run a high risk of developing pulmonary edema, where as the patients with asymptomatic valvular insufficiency tend to tolerate volumetric overload during pregnancy much better.^6^

To improve care further we need to know why pregnant women are still dying, it is therefore important to identify strategies to reduce maternal deaths due to cardiac disease in pregnant women.^7^ Presence of valvular heart disease in pregnant women is a risk to the fetal well being. Pregnant women with valvular heart disease and good cardiac status during pregnancy have been shown to develop less intrauterine growth retardation, less premature births and less maternal mortality and morbidity.^8^

These patients must be managed with a multidisciplinary approach, with the collaboration of an obstetrician and cardiologist. Furthermore, this management must commence before conception, the family should be counselled about the possible risks and optimal conditions must be maintained for conception.^9^ If possible, pregnancy should not be allowed in patients with uncorrected severe valvular lesion or those requiring anticoagulation. This is due to increased maternal and fetal morbidity and mortality. Medical therapy and balloon valvuloplasty have greatly improved the outcome and now term gestation is possible.^2^ Identification of pregnancy related cardiac and neonatal complications is important. It is equally important to identify prior risk factors that are capable of predicting the likelihood of adverse pregnancy outcome.^10^

The aim of study is to evaluate the frequency of fetomaternal outcome of pregnancy with Mitral Stenosis. This study provides local data and describe the burden of disease thereby helping in development of a patient specific management plan.

## METHODS

This descriptive, case-series study was conducted in the department of obstetrics and gynaecology, Civil Hospital, Karachi from 1^st^ January 2011 to 31^st^ December 2012. One hundred and one patients admitted in Department of obstetrics and Gynaecology, Civil Hospital, Karachi were included following non probability, convenient sampling.

### Inclusion Criteria

All women with Mitral stenosis (mild, moderate and severe) diagnosed on echocardiography after first trimester (after 12 weeks of gestation calculated by ultrasonography).

### Exclusion Criteria

All women with other medical disorders, e.g. anemia, congenital heart diseases, cardiomyopathy hypertension, asthma, diabetes mellitus, renal disease, thyroid disease, Smokers.

### Data Collection

Women admitted with Mitral stenosis were enrolled in the study. The purpose, procedure of the study was explained and informed consent was taken. History was taken regarding age, parity, complaints like breathlessness, generalized weakness, palpitations. Gestational age was calculated by ultrasonography. All patients were advised Echocardiography for diagnosis and severity of Mitral stenosis. Assessment of patient for new onset heart failure and Ejection fraction were recorded. Mode of delivery was observed. Maternal death was noted. Fetal outcome was assessed in terms of low APGAR score at five minutes, low birth weight, prematurity (gestational age<37) and intrauterine death. This information was entered in proforma.

### Data Analysis

SPSS version 10 was used for data analysis. Mean ± SD was calculated for age, parity, gestational age, low APGAR score (<7 at five minutes), low birth weight (<2.5 kg), ejection fraction. Frequency and percentages were calculated for patients with mild, moderate and severe mitral stenosis, patients who develop heart failure, patients who expired, mode of delivery, and foetal outcome like intrauterine death, low birth weight, prematurity. Stratification was done with regards to age, parity, booking status and severity of Mitral stenosis to see the effect of these on outcome.

## RESULTS

A total of 101 pregnant women at 13 – 42 weeks of gestation with mitral stenosis (mitral valve area ≤ 2.5 cm^2^) were included in the study. Mean ± SD age of patients was 26.3 ± 4.3 years. Majority of women 70 (69.3%) were between 20 – 29 years of age. Among 101 women, 82 (81.2%) women were multiparous, while 19 (18.8%) women were primigravida. Forty two (41.6%) women were booked and 59 (58.4%) were unbooked. Mean ±SD gestational age at birth was 36.3 ±2.8 weeks. Pre-maturity (< 37 weeks of gestation) was found in 22 (21.8%) case. Table-I

**Table-I:** Demographic data of patients n=101.

Variable	No	Percentage
*Age*		
20-29 yrs	70	69.3%
>29 yrs	31	30.7%
*Parity*		
PG	19	18.8%
1	31	30.7%
>2	51	50.5%
*Booking Status*		
Un-booked	59	58.4%
Booked	42	41.6%
*Gestational Age (Weeks)*		
<37	22	21.8%
>37	79	78.3%

Vaginal delivery was the most common mode of delivery found in 67 (66.3%) women followed by lower segment caesarean (LSCS) in 34 (33.7%) women. Mean ± SD birth weight of neonates was 2.6 ± 0.52 kg. Low birth weight (<2.5kg) was found in 28(27.7%) neonates. Mean ± SD APGAR score was 6.99 ± 0.9. APGAR score < 7 was found in 15 (14.9%) neonates. Intrauterine death occurred in 9 (8.9%) of cases. Maternal mortality was 1.9%. Table-II

**Table-II:** Maternal and Fetal Outcome n= 101.

	No. of women	Percentage
*Modes of Delivery*		
Vaginal	67	66.3
LSCS	34	33.7
*Birth Weight*		
Low = (<2.5kg)	28	27.7
Normal	73	72.3
*APGAR Score (At 5min)*		
≥7	85	85.1
<7	15	14.9
*Neonatal outcome*		
IUD	9	8.9
LIVE	92	91.9
*Maternal mortality*	2	1.9

Moderate stenosis were found in 26 (25.7%) women and severe stenosis in 14 (13.9%) women. New onset of heart failure was diagnosed on echocardiography. Low ejection fraction (less than 55%) was diagnosed in 20 (13.8%) women. Table-III Out of twenty women, 10 (50%) women with severe Mitral stenosis had ejection fraction <55%, while 9 (45%) women with moderate stenosis had low ejection fraction. Proportion of low ejection fraction was high in multiparous women and in women between 20-29 years of age. Fourteen (73.7%) of non booked cases had low ejection fraction. Table-IV

**Table-III:** Grading of Mitral Stenosis n=101.

Mitral Stenossis	No. of Women	Percentage
Mild	61	60.4
Moderate	26	25.7
Severe	14	13.9
*Ejection Fraction*		
Low (<55%)	20	19.8
(>55%)	81	80.2

**Table-IV:** Low ejection fraction (<55%) with respect to mitral stenosis, age, parity and booking status. n=20

	Ejection Fraction (<55%)	Percentage
*Mitral Stenosis*		
Mild	1	5
Moderate	9	45
Severe	10	50
*Age (years)*		
20-29	15	75
>29	5	25
*Parity*		
0	8	40
1	3	15
≥2	9	45
*Booking Status*		
Booked	6	30
Non booked	14	70

Proportion of low APGAR score, low birth weight and pre-maturity were high in mild mitral stenosis. Women with mild Mitral stenosis had premature babies in 12(54.5%) cases, APGAR score <7 in 8 (53.3%) cases and 14(50%) neonates had low birth weight. Frequency of intra uterine death (IUD) was high in severe mitral stenosis. Table-V

**Table-V:** Fetal outcome with respect to Mitral stenosis n=101.

Mitral Stenosis	Low Apgar score (<7)	Low birth weight (< 2.5 kg)	Pre-Maturity (<37 weeks)	IUD
Mild	8 (53.3%)	14(50%)	12(54.5%)	2(22.2%)
Moderate	4 (26.7%)	7 (25%)	5 (22.7%)	3 (33.3%)
Severe	3 (20%)	7 (25%)	5 (22.7%)	4 (44.5%)

## DISCUSSION

Management of pregnancy in patients with valvular heart disease (VHD) continues to pose a challenge to the clinician. Although the risk in such patients have been recognized, they have not been well defined as available information is based mostly on anecdotal reports or small series of patients without an appropriate control population.^1^

Heart diseases are the most important non obstetrical causes of maternal deaths during pregnancy, accounting for almost 10% of maternal deaths. They complicate 1-3% of all pregnancies with congenital defects in 70-80% of the cases.^2^ Mitral stenosis is most commonly secondary to acute rheumatic fever.^11^ Foetal mortality is not exceptionally high in patients with New York Heart Association (NYHA) class I and II, however if there is associated pulmonary hypertension, risk of abortions, intrauterine growth retardation, preterm delivery and early neonatal death^5^ is high.

Child bearing women with cardiac disease present a unique challenge to the health care provider. The physiological adaption or pregnancy predisposes cardiac patient to decompensate. Classic symptoms of heart disease mimic common symptoms of pregnancy. Detail assessment of patient throughout pregnancy may lead to initial discovery of heart disease.^6^ Pregnancy in women with Mitral valvular stenosis is associated with marked increase in maternal morbidity.^12^ The high Maternal morbidity and mortality is due to inability to cope with physiological adaptation of pregnancy, stress of labor and hemodynamic changes of puerperium.^13^ If diagnosed early, and managed properly with multi disciplinary approach, it results in successful outcome for mother and child in majority of cases.^14^ Echocardiography in pregnancy is the imaging tool used to assess severity of cardiac disease.^15^

In this study mild mitral stenosis was found in 60.4% women, moderate stenosis in 25.7% and severe in 13.9% of women. Vaginal delivery was the most common mode of delivery found in 66.3% women followed by lower segment caesarean section (LSCS) in 33.7% women, which is in accordance with the result of 13% in a study by Avila et al.^16^

A study done by Hameed et al.^17^, showed mode of delivery was vaginal in 61 (92%) out of 66 patients with valvular heart disease and others had cesarean section due to obstetric indication and cardiac lesions. Another study done by Bonow et al.^18^ showed mode of delivery was vaginal in 196(78.1%) out of 251 and cesarean section done on 55 (21.9%) patients.

Fetal outcome depends on the degree of maternal well being and gestational age. Cardiac patients have babies lighter by about 200gm. Mean ± SD birth weight of neonates was 2.6 ±0.52kg. Low birth weight (<2.5kg) was seen in 27.7% neonates. Mean ± SD gestational age at birth was 36.3 ± 2.8 weeks. Pre-maturity was found in 22 (21.8%) cases. A local study^2^ in Pakistan showed 14% cardiac women had preterm deliveries and 42.5% were of low birth weight. Intrauterine death occurred in 8.9% of cases.

Maternal mortality generally varies directly with functional classification at pregnancy onset. There were two maternal deaths comparable with the study done by Wasim T^19^ which showed 3.8% maternal mortality.

A study done by Sawhney et al.^20^, showed that live births were 252 out of 254 patients and two were stillbirths, mean (±SD) birth weight was 2.6_±_kg. Another study^17^ showed that live birth was 64 out of 66 patients and still birth was two. Preterm delivery was 15 (23%) out of 66. Studies done by Hsich et al.^9^ and Sermer M^10^ reported higher incidences of preterm birth in patients with valvular heart disease. A study done by Ashwani^21^ showed 51.3% incidence of Mitral stenosis. Spontaneous vaginal delivery was seen in 50%, instrumental deliveries in 16.6% and cesarean section in 28.4%. Maternal mortality was 3.3% and perinatal mortality 6.6%.

Pregnancy in our patients was associated with an increased incidence of IUGR, preterm deliveries and lower birth weight, especially in cases with moderate and severe Mitral stenosis. Hemodynamic compromise secondary to valvular stenosis and the resulting decrease in uterine blood flow are probable explanations for the high incidence of impaired intrauterine fetal growth seen in this study. The importance of prepregnancy diagnosis, counseling and contraception are essential to safely manage the planned pregnancies in cardiac patients.^22^

## CONCLUSION

Heart disease in pregnancy is associated with significant morbidity, it should be carefully managed in a tertiary care hospital to obtain optimum maternal and fetal outcome.

## RECOMMENDATIONS

Cardiac disease during pregnancy continues to be a major health problem, especially in developing countries. Some recommendations to reduce the fetomaternal morbidity and mortality are:

- Multidisciplinary approach involving a cardiologist, neonatologist and obstetricians.
- Raising the status of government health care facilities where less privileged women have access.
- Adequate training of health care of workers in the identification of symptoms and signs of cardiac disease for early detection and referral.
- Routine examination of the cardiovascular system in the antenatal clinic is essential for early diagnosis and appropriate management.
- Once identified, a pregnant woman with cardiac disease must follow secondary and tertiary levels of care by specialized units.
- Pre-pregnancy counselling.
- Particular attention need to be paid on contraception and future pregnancies.
- Close surveillance during pregnancy. Monitoring should continue after delivery.
- Adequate and timely follow up.
